# Zooarchaeology through the lens of collagen fingerprinting at Denisova Cave

**DOI:** 10.1038/s41598-021-94731-2

**Published:** 2021-07-29

**Authors:** Samantha Brown, Naihui Wang, Annette Oertle, Maxim B. Kozlikin, Michael V. Shunkov, Anatoly P. Derevianko, Daniel Comeskey, Blair Jope-Street, Virginia L. Harvey, Manasij Pal Chowdhury, Michael Buckley, Thomas Higham, Katerina Douka

**Affiliations:** 1 Institute for Scientific Archaeology, Eberhard Karls University of Tübingen, Tübingen, Germany; 2 Max Planck Institute for the Science of Human History, Jena, Germany; 3 Institute of Archeology and Ethnography, Siberian Branch of the Russian Academy of Sciences, Novosibirsk, Russia; 4 Oxford Radiocarbon Accelerator Unit, RLAHA, University of Oxford, Oxford, OX1 3QY UK; 5 Department of Earth and Environmental Sciences, School of Natural Sciences, The University of Manchester, Manchester, M13 9PL UK; 6 Manchester Institute of Biotechnology, The University of Manchester, Manchester, M1 7DN UK; 7 Department of Evolutionary Anthropology, Faculty of Life Sciences, University of Vienna, Vienna, Austria

**Keywords:** Archaeology, Palaeontology

## Abstract

Denisova Cave, a Pleistocene site in the Altai Mountains of Russian Siberia, has yielded significant fossil and lithic evidence for the Pleistocene in Northern Asia. Abundant animal and human bones have been discovered at the site, however, these tend to be highly fragmented, necessitating new approaches to identifying important hominin and faunal fossils. Here we report the results for 8253 bone fragments using ZooMS. Through the integration of this new ZooMS-based data with the previously published macroscopically-identified fauna we aim to create a holistic picture of the zooarchaeological record of the site. We identify trends associated with climate variability throughout the Middle and Upper Pleistocene as well as patterns explaining the process of bone fragmentation. Where morphological analysis of bones from the site have identified a high proportion of carnivore bones (30.2%), we find that these account for only 7.6% of the ZooMS assemblage, with large mammals between 3 and 5 more abundant overall. Our analysis suggests a cyclical pattern in fragmentation of bones which sees initial fragmentation by hominins using percussive tools and secondary carnivore action, such as gnawing and digestion, likely furthering the initial human-induced fragmentation.

## Introduction

Studying highly fragmented archaeological and paleontological bone assemblages is a particularly challenging task. Robust enough that they can survive a range of depositional environments, bones are still susceptible to numerous taphonomic processes^1–4^. Research into large assemblages of fragmented bones has been instrumental in exploring depositional histories and accumulation^5–8^ and the impact of freezing and thawing^9^, weathering^10,11^, trampling and gnawing^12^, and the role of humans modifying and processing bones, either for subsistence reasons (e.g. bone marrow exploitation and fuel use)^13–16^ or for the production of osseous tools^17,18^. Fragmentation adversely affects taxonomic and anatomical attribution and it is estimated that only a third of bones found in Pleistocene archaeological contexts can be identified using traditional methodologies, i.e. through visual inspection^19,20^. Without a reliable means of determining taxonomically highly fragmented bones these are often recorded as “unidentified” fauna excluding their integration into detailed zooarchaeological studies and the possibility of identifying new faunal groups and uncommon taxa.

Recently the potential of highly fragmented Pleistocene-age bone assemblages has been explored through the use of peptide mass fingerprinting, specifically using ZooMS (Zooarchaeology by Mass Spectrometry)^21^. ZooMS provides an efficient means of screening and taxonomically identifying bones through the targeted analysis of type I collagen (COL1). The method has been applied to large Pleistocene assemblages^22–24^, including the Middle and Late Pleistocene site, Denisova Cave, in the Russian Altai^25,26^. ZooMS analysis of fragmented bones at Denisova Cave has so far resulted in the identification of nine new hominin remains^25–27^, including two Neanderthals (Denisova 15 and Denisova 17), three Denisovans (Denisova 19, Denisova 20, and Denisova 21), and the offspring of a Neanderthal mother and a Denisovan father (Denisova 11)^25,28^. In the course of identifying these individuals a large assemblage of fauna has been identified which has not been discussed thus far.

Here we report the results of 8253 fragmented bones from Denisova Cave that were analysed using ZooMS. These bones were initially studied with the aim of identifying hominin remains and, in light of recent publications detailing the chronology of the site^26,29^, zooarchaeological findings^29–34^, and palaeoenvironmental reconstructions^35,36^ we aim to identify what contribution, if any, these fragmented bones may play in our understanding of Denisova Cave’s faunal record by reincorporating them into research at the site.

The addition of ZooMS-based determinations into the more traditional zooarchaeological analysis is inherently complicated, particularly when analysing morphologically non-diagnostic bones. A first limitation stems from the broad taxonomic groupings created through ZooMS analysis which do not match the often more detailed macroscopic identifications. For large mammals, ZooMS is typically only able to make genus level identifications, with some exceptions in which species can be determined^37^. Second, because NISP (Number of Individual/Identified Specimens) is the only reliable measurement through which these highly fragmentary bones can be counted, this inevitably leads to an overestimation of the presence/absence of fauna^19,20^. With these limitations in mind, and using different approaches we detail below, we use ZooMS-identified bones to create a holistic view of the fauna assemblage from Denisova Cave and determine broad trends and variation through time.

## Materials

Denisova Cave, situated in the Altai Mountains of Russian Siberia, is unique in its hominin record as the only site where both Neanderthal and Denisovan fossils have been identified (Supplementary Fig. 1)^38–41^. The cave, situated in the ledge of a southwest-facing rock wall and overlooking the Anui River, has the longest stratigraphic sequence for northern Eurasia, with contexts dating from the Middle Pleistocene. This extensive sequence has made Denisova Cave a type-site for northern Eurasia, allowing for palynological^42^ and palaeoecological^43,44^ reconstructions of the region and several zooarchaeological studies^29–34,36^. Despite particularly favorable conditions that ensure a high degree of biomolecular preservation at the site, less than 5% of bones excavated can be identified macroscopically^29–34,36^, making Denisova Cave cave a good candidate for the application of ZooMS. We analysed 8,253 non-diagnostic bone fragments which were excavated from each of the three interconnecting chambers of Denisova Cave. The majority of these bones come from the East Chamber (n = 6288), followed by the Main (n = 1143) and South (n = 822) chambers (Supplementary Information; Supplementary Fig. 1). These were sampled with the permission of and in collaboration with the Institute of Archeology and Ethnography of the Siberian Branch of the Russian Academy of Sciences, Novosibirsk, Russia.

Bones were specifically chosen as they could not be identified on the basis of morphology and, where possible, fragments > 2 cm in length were preferentially selected, although smaller bones were also part of our analysed assemblage. Assemblages of bones of this size were also assumed to contain morphologically indistinct hominin remains since all previously identified hominins at the site were no bigger than ~ 3 cm in length. Samples were drilled in preparation for analysis and from each bone approximately 20 mg was removed using a diamond covered disc. Care was taken to clean equipment between drilling samples to minimise cross-contamination. Sampling was undertaken at two laboratories, at the School of Archaeology, University of Oxford, U.K. and at the Department of Archaeology, Max Planck Institute for the Science of Human History (MPI-SHH), Jena, Germany.

## Results

**Figure 1:**
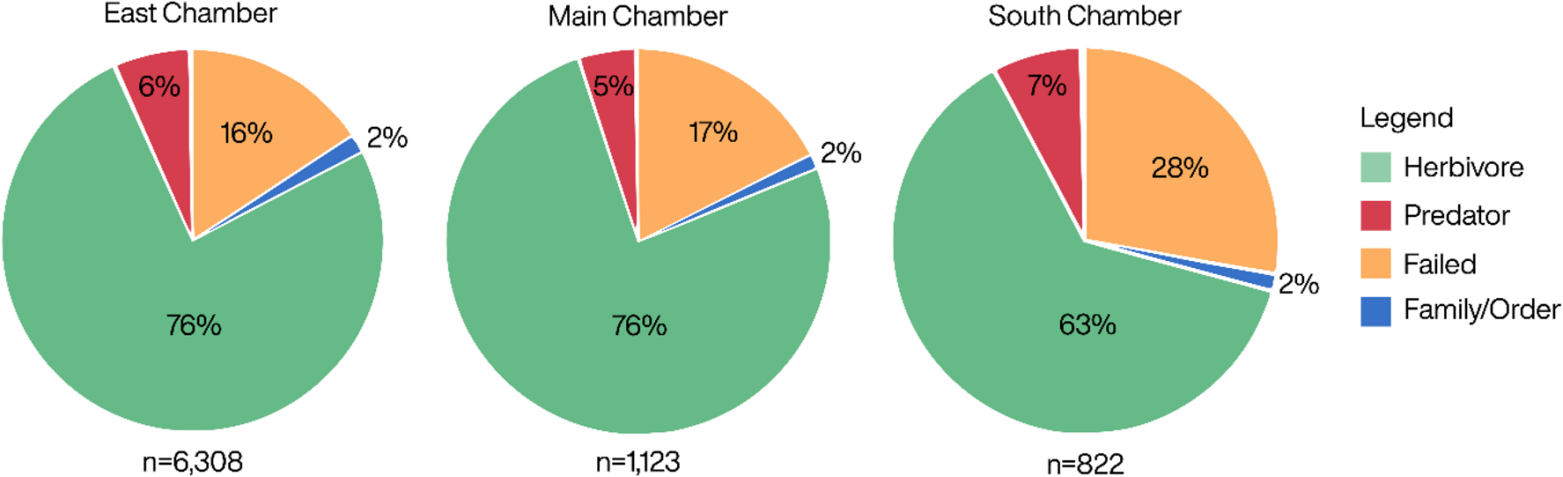
Comparison of overall ZooMS results by chamber of Denisova Cave showing samples which could be identified to their most specific ZooMS taxon (shown here as herbivores and predators) in comparison with samples which could only be identified to family/order or those which failed analysis.

Overall, of the 8253 analysed bones, 74% were assigned to a specific ZooMS taxon whereas an additional 5% could only be identified to family or order level due to low quality spectra (Supplementary Information). The South Chamber had a high rate of samples that failed to produce enough collagen for taxonomic identification (28%), in comparison with lower rates of failure for the East (16%) and Main (17%) chambers (Fig. 1).

In total we identified 18 ZooMS taxa amongst the bones we analysed (Table 1). A ZooMS taxon is defined here as the most specific identification ZooMS is able to provide, generally to a genus or family level. For instance, several Canids have been identified within the morphological assemblage at Denisova Cave, including *Canis lupus* (wolf), *Vulpes corsak* (corsak fox), *Cuon alpinus* (dhole), and *Alopex lagopus* (arctic fox), all of which could only be classified as ‘Canidae’ through ZooMS analysis as their peptide mass fingerprints are identical. *Vulpes vulpes* (red fox) is the only Canid that can be differentiated using ZooMS, as a result of a small difference in their COL1ɑ2 484‒498 peptide^45^ (Supplementary Table 1). The assemblage is dominated by large vertebrates, with small vertebrates and birds accounting for less than 1% of the identified material examined. This is likely in part a result of our selection of bones which were preferentially 2 cm in length or more.

**Table 1:** The results of ZooMS analysis of 8,253 fragmented bones from Denisova Cave.

ZooMS taxon	East chamber	Main chamber	South chamber	Total
Bison/Yak	1629	337	221	2187
Canidae	140	12	8	160
Capra	209	75	30	314
Cervidae	18			18
Cervidae/Gazella/Saiga	1189	35	32	1256
Crocuta/Panthera	99	21	19	139
Elephantidae	181	40	35	256
Equidae	669	123	58	850
Felidae	7	1		8
Hominin	6	1		7
Leporidae	2			2
Muridae	2			2
Mustelidae	2			2
Ovis	147	108	40	295
Ovis/Capra	302	67	54	423
Rangifer	34	3	1	38
Rhinocerotidae	401	98	48	547
Ursidae	123	20	33	176
Vulpes vulpes	19			19
Bird	9	1		10
Capra/Rangifer	12	2	3	17
Cervidae/Gazella/Saiga/Equidae	15		1	16
Crocuta/Panthera/Mustelidae	11	5	4	20
Equidae/Rhinocerotidae		1		1
Felidae/Crocuta/Panthera	1			1
Felidae/Crocuta/Panthera/Mustelidae	2			2
Felidae/Ursidae	18	4	1	23
Ovis/Capra/Cervidae/Gazella/Saiga	25			25
Ovis/Capra/Rangifer	4		2	6
Ovis/Cervidae/Gazella/Saiga	25	3		28
Unknown	19	3	3	25
Failed	968	183	229	1380
Total	6288	1143	822	8253

For the species most likely included in the “ZooMS-taxon” see Table S1.

### Comparing datasets

**Figure 2:**
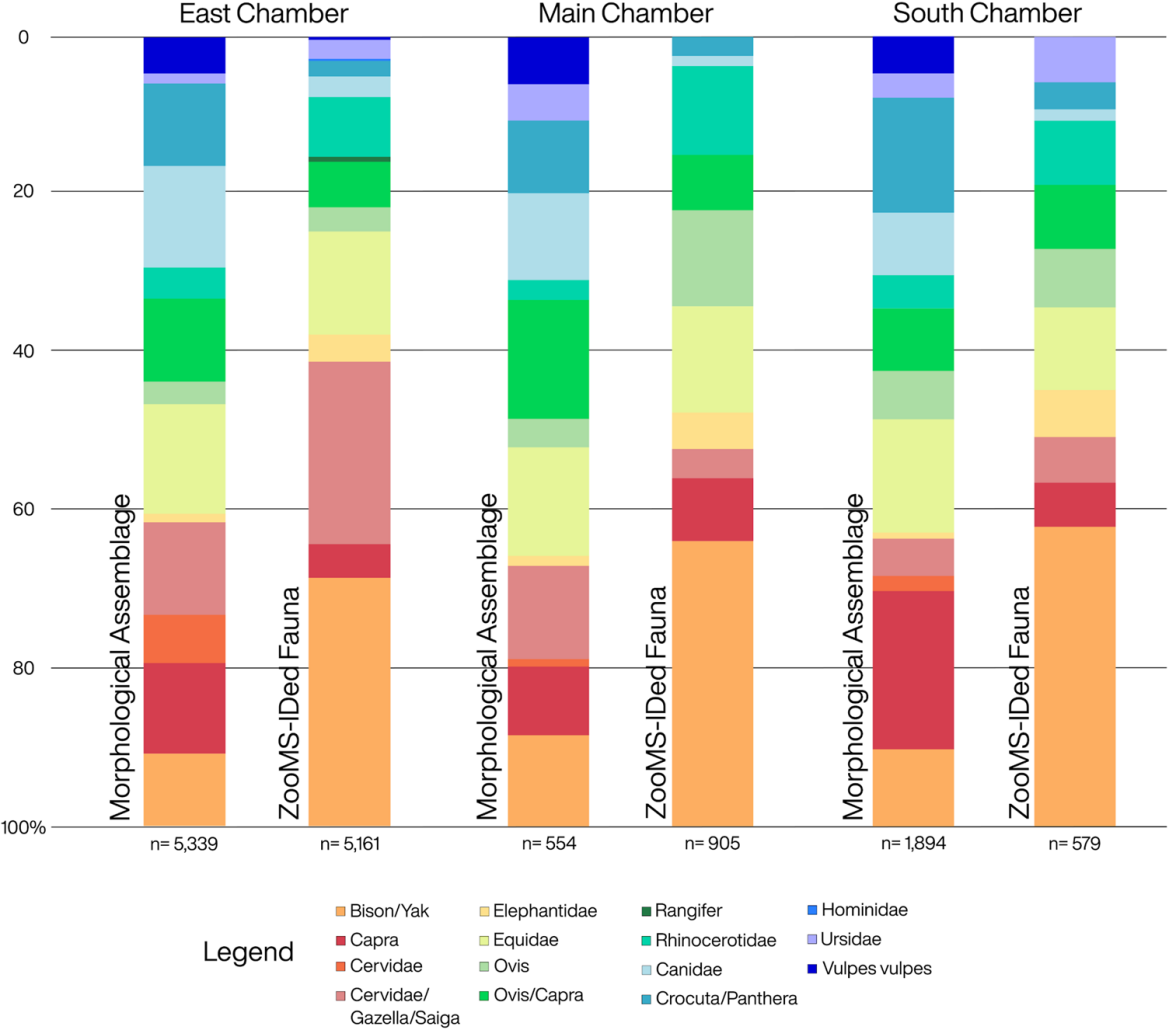
Comparison of the overall abundance of fauna between the morphological assemblage and the ZooMS-IDed fauna for the three chambers of Denisova Cave. Only the largest faunal groups have been included. Small mammals from both the morphological assemblage and ZooMS-IDed component, samples which failed ZooMS analysis, and “Unknown” spectra are not included.

In order to compare the ZooMS identified fauna with previously-published zooarchaeological datasets from Denisova Cave we have compiled those datasets, referred to as the ‘morphological assemblage’, from the literature and converted them into ZooMS-style groupings (following Supplementary Table 1) which can then be directly compared with the new ZooMS results we present here (Fig. 2). In our discussion of percentages, we have excluded samples which failed ZooMS analysis or those which could not be assigned to the most specific ZooMS taxon possible, with the exception of Ovis/Capra and Crocuta/Panthera (see Supplementary Information) in order to make them more compatible with zooarchaeological results. We have also excluded small vertebrates as they were not identified in sufficient numbers for direct comparison with the morphological assemblage. While some results are not included in our comparisons with the zooarchaeological datasets, all results are reported in the Supplementary Information (Supplementary Table 2, 3, 4; External Database 1).

We use the term “predator”, rather than “carnivore”, to group the activity of hominins, Canidae, Crocuta/Panthera, Ursidae and *V. vulpes*, some of which have varied diets. The term “herbivores” includes Bison/Yak, Capra, Cervidae/Gazella/Saiga, Elephantidae, Equidae, Ovis, and Rhinocerotidae.

### East chamber

ZooMS analysis was carried out on 6,288 bone fragments from most archaeological layers of the East Chamber (Table 1; Supplementary Table 2). Predators account for 20% of the morphological assemblage of the East Chamber, in comparison with only 7.7% of the ZooMS-IDed assemblage. The overall proportion of predator remains does not vary significantly throughout the layers of the East Chamber in the morphological assemblage. Cave hyaena (*Crocuta crocuta spelaea*) are the dominant predators between layers 9–13, alongside smaller numbers of Panthera, Canidae, and Felidae^29,31^. Their behaviours are particularly visible in the archaeological material from layers 9–11 where a large number of bones have clearly passed through the digestive tracts of hyaenas or wolves, leaving acid corrosion marks on bones and dissolving the enamel on teeth^29,31^. In contrast, in the ZooMS-IDed component, Crocuta/Panthera are present in relatively low numbers, accounting for approximately 1.9% of all identified bones (Fig. 2). Canidae on the other hand are consistently the dominant predator group in the ZooMS-IDed component for each of the studied layers of the East Chamber. Within the morphological assemblage, Canidae only exceed Crocuta/Panthera in layers 15–17^29,31^ a period during which forest species like *Capreolus pygargus* (Siberian roe deer) and *Cervus elaphus* (red deer) are the major herbivore groups present in the assemblage (see below). In the ZooMS-IDed fauna, Canidae account for 2.7% of all the bones identified within the East Chamber.

The two most significant herbivore taxa in the ZooMS-IDed component for the East Chamber are Cervidae/Gazella/Saiga and Bison/Yak. The Cervidae/Gazella/Saiga ZooMS-taxon includes *C. pygargus*, *C. elaphus*, *Megaloceros giganteus* (Irish elk), *Alces alces* (elk), *Saiga tatarica borealis* (saiga antelope), and *Procapra gutturosa* (Mongolian gazelle). The only cervid species present at Denisova Cave which can be separated from this group is the Siberian roe deer on the basis of their COL1ɑ2 757–789 (ɑ2 757)^45^ peptide marker. The ɑ2 757 peptide for Siberian roe deer is present at *m/z* 3043.4/3059.4 but at *m/z* 3017.5/3033.5 for Cervidae/Gazella/Saiga. Since this differentiation is based on a single marker it is possible that many of the generically identified Cervidae/Gazella/Saiga are actually Siberian roe deer which were missing their ɑ2 757 marker in poorer preserved specimens. The ɑ2 757 marker is easily lost as a result of collagen degradation, a common problem for instance in the separation of *Ovis* and *Capra* which is done on the basis of the same peptide^46^.

**Figure 3:**
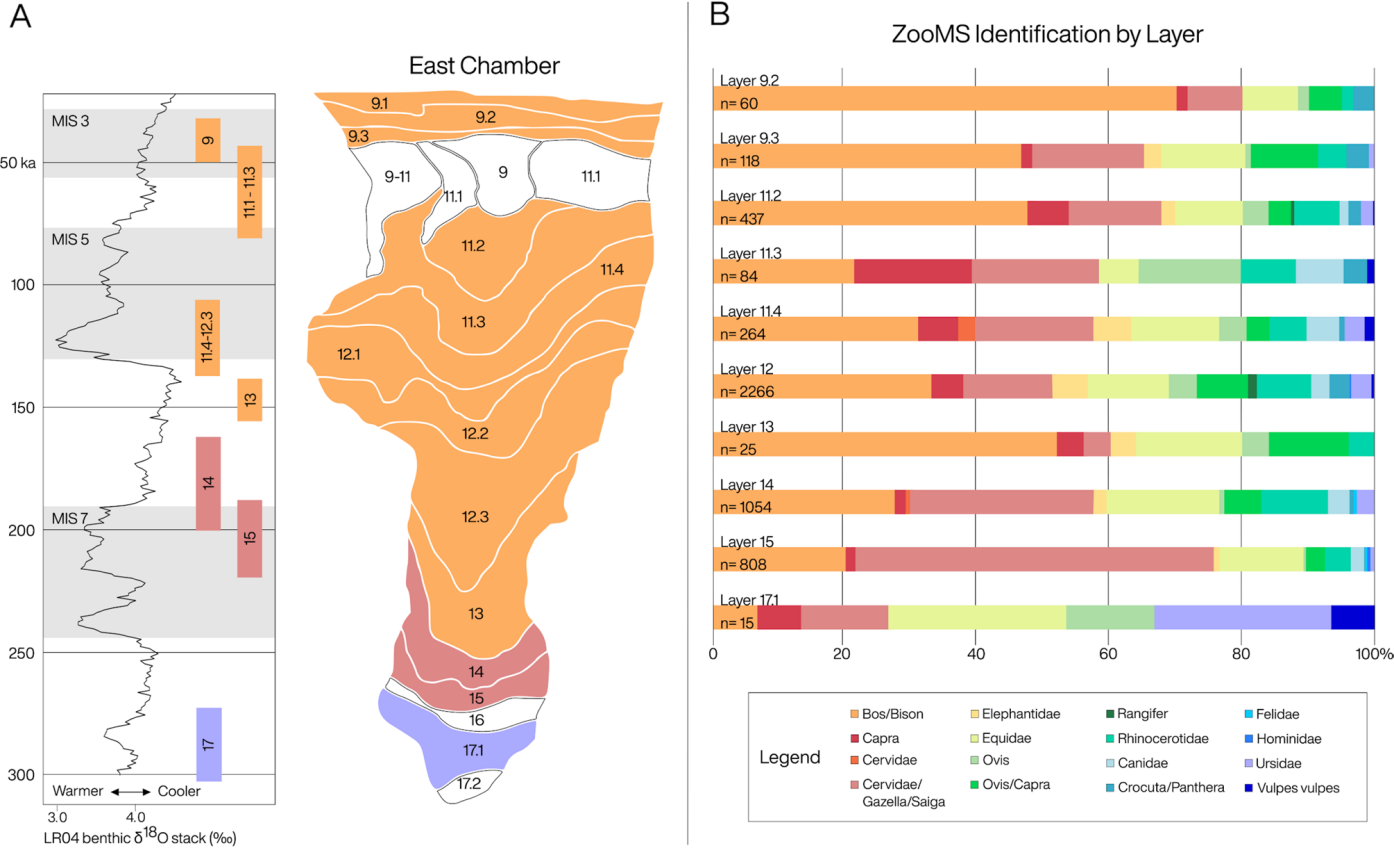
Relative abundance of fauna identified using ZooMS per layer of the East Chamber of Denisova Cave. This information can also be found in Supplementary Table 2. (**A)** The stratigraphy and corresponding age ranges are highlighted with the most abundant fauna for each layer. The marine-oxygen isotope curve was compiled from benthic δ18O records^64^; marine-oxygen isotope stages 3, 5, and 7 are highlighted in grey^29^. (**B)** Each bar depicts the major taxa identified. Only the largest groups of fauna have been included, excluding small mammals, “Unknown” identifications, and failed samples.

Cervidae/Gazella/Saiga bones account for 54% of the ZooMS-IDed component for layer 15. This number declines significantly in the overlying archaeological layers. In comparison, Bison/Yak, a taxonomic group that contains the remains of steppe bison (*Bison priscus*) and the less common Baikal yak (*Poёphagus mutus*), continue to increase by percentage throughout the stratigraphic layers. Bison/Yak eventually account for 70% of the ZooMS IDed fauna in layer 9.2^26^ (Fig. 3; Supplementary Table 2).

### Main chamber

We carried out ZooMS analysis for 1,143 bones from layers 9–12 of the Main Chamber (Table 1; Supplementary Table 3). Predators dominate the morphological assemblage, accounting for more than 30% of all identifiable bone for layers 9–12. In comparison, the dominant predators, Canidae, Crocuta/Panthera, and Ursidae, make up only 5.5% of the ZooMS-IDed component. Our Crocuta/Panthera taxon likely includes *C. spelaea*, although *Panthera spelaea* are also present in the earliest Pleistocene layers of the Main Chamber. Crocuta/Panthera dominate the morphological assemblage for layers 9–11, accounting for 9.4% of identifiable bones (Fig. 2)^29^. In the ZooMS-IDed component, Crocuta/Panthera account for 1.68% of the fragmented bones for layers 9–11 making them the most commonly identified predators for these contexts. The morphological assemblage identifies Canidae as the major predator group in layer 12^29,34^, however none were identified in the ZooMS-IDed component for this context.

The major herbivore groups do not differ significantly between the morphological and ZooMS identified fauna. Equidae, Ovis/Capra, and Cervidae/Gazella/Saiga are present in relatively large numbers in each dataset. Bison/Yak remains increase significantly within the ZooMS-IDed fauna, accounting for 35% of the assemblage, more than four times the amount identified within the morphological bones (8.35%).

### South chamber

We analysed 822 bones from the South Chamber using ZooMS (Table 1; Supplementary Table 4). Crocuta/Panthera, Canidae, and Ursidae are the dominant predator groups in the ZooMS fauna in this Chamber. They account, however, for only 10.5% of this assemblage, three times less than predators in the morphological assemblage (Fig. 2). Ursidae (*Ursus (Spelaearctos) savini*) account for 9.7% of the ZooMS-IDed fauna for layer 11. Previous zooarchaeological analysis suggested the cave may have been used for hibernation by the small cave bear (*U. spelaearctos*)^32^ which could explain the unusually high number of bear bones. Crocuta/Panthera were the dominant predator group for the ZooMS-IDed fauna in layer 12, accounting for 2.7% of the assemblage which is largely in line with dominant predator groups identified in the morphological assemblage. Bison/Yak (36.6%) and Equidae (10.3%) were the most common herbivores present within the ZooMS-IDed component.

Zooarchaeological analysis of bones from the South Chamber^32,33^ suggests intense predator presence for layers 9–12, in particular Crocuta/Panthera. Their bones are disproportionately represented in the morphologically identifiable bones for South Chamber (n = 255), second only to *Capra* sp. remains, which are identified as the likely prey for cave hyena and snow leopard^32,33^. Fragments of cave hyena coprolites were identified throughout layers 9–12 of the South Chamber and many of the bones of prey taxa show traces of acid corrosion, indicating that they have passed through the digestive tracts of predators^32,33^. It is noteworthy that only 3.7% of bones recovered from this part of the cave are larger than 5 cm^33^. We may hypothesize that this large degree of fragmentation is due to predation. In turn, such a high concentration of carnivore processing could explain the low level of protein preservation in this section of the cave; 28% of the fragmented remains analysed using ZooMS failed to produce enough collagen for identification as opposed to 17% and 16% for the East and Main Chambers, respectively (Fig. 1; Table 1; Supplementary Table 4).

## Discussion

### Patterns of faunal variability

ZooMS has previously been applied to larger microfauna assemblages and older individual faunal samples^47,48^, yet our present work is the largest application of ZooMS to an assemblage of fragmented bones with an archaeological association. The oldest archaeological layers we study here (layers 15 and 14) date to between 217 and 163 ka^29^. For layer 15, from which we analysed nearly 1000 bones, the success rate was 86% (Supplementary Table 2). This is particularly encouraging and paves the way for the future analysis of bone assemblages of similar biomolecular preservation and antiquity.

Significant differences in success rates were observed across layers and the three non-connecting chambers. Bones from the East Chamber were the most successful, however an average of 35% of samples from the youngest Pleistocene contexts (layers 9.2 and 9.3) failed to produce enough collagen for ZooMS analysis. This high failure rate is likely due to the heavy phosphatisation in the soil^36,49^ which has previously impeded OSL, radiocarbon dating, and genetic analyses for these layers^26,29^. At present, it seems identifying fossils suitable for biomolecular analysis from Upper Pleistocene contexts at Denisova Cave will continue to be challenging. This is unfortunate given the persistent questions regarding which hominin populations were present in the Altai Mountains during this period^26^.

At Denisova Cave a high degree of bone fragmentation is observed overall. Of the 177,000 bones excavated at the East Chamber more than 95% were less than 2–5 cm in length and could not be identified macroscopically^29,31^. Using ZooMS however, on average 74% of the fragmented bones we analysed could be assigned to a specific ZooMS taxon and an additional 5% of samples that produced low quality spectra could be identified to family or order levels. This contrast in identification success (< 5% identified morphologically versus ~ 80% based on ZooMS) highlights once again the merits of large-scale application of ZooMS on highly fragmented archaeological assemblages.

Bones analysed in this study from the Main Chamber were selected from the Upper Palaeolithic layers 9 and 11 (Supplementary Fig. 1), whereas bones from the South Chamber came from both Upper Palaeolithic layer 11 and Middle Palaeolithic layers 12 and 21. For the East Chamber, bones were selected from all major stratigraphic units, from the early Middle Palaeolithic through to the Upper Palaeolithic layers (15–9), and the archaeologically sterile layer 17. The majority of bones from the ZooMS-IDed component from all three chambers were the remains of prey, suggesting that the formation of the assemblage is not entirely relegated to equifinality in that some processes of the accumulation can be unravelled^3,4^. In the East Chamber, the earliest evidence for hominins processing carcasses and modifying bone is present in layers 15 and 14 of the East Chamber which coincides with the arrival of the first Denisovans to the site^27,35,50^. From this period lithic technologies are continuously present throughout the contexts of this chamber and the layers studied for the Main and South chambers^35,51^. Alongside hominins, previous zooarchaeological studies for Denisova Cave have highlighted the major role carnivores likely played in the formation of the site’s fragmented bone assemblage^30,33^. Carnivore remains are found in all contexts for the site but their behaviours are particularly visible in Upper Palaeolithic contexts with extensive gnawing marks and digestion of bones likely by hyenas, panthers, leopards, and wolves^31,33,35^. The overlap and relationship between hominins and predators at Denisova Cave has been investigated through multiple studies. While periods of intensive hominin presence are evident, particularly during the Early Middle and Middle Palaeolithic contexts where anthropogenic marks are frequently found on bones^29,31^, evidence for other predators at the site is continuously documented throughout the Palaeolithic^30–33^.

Recent analysis of the microstratigraphy of Denisova Cave has furthered this, showing that due to slow sedimentation accumulation rates it is currently not possible to identify when predators like bears, wolves, and hyaenas were occupying the cave as opposed to hominin groups like Denisovans and Neanderthals^36^. Rather, it seems that many groups were alternating as the site’s main occupants throughout the Pleistocene^32,36^. Further analyses of the bone assemblage, such as detailed taphonomic studies, bone refitting exercises and extensive dating of specific locations to assess the contemporaneity of various species at the site, will shed further light on this. Early evidence however suggests that at times hominins and other predators may have even benefited from each other’s presence. The close stratigraphic relationship between carnivores and hominins at Denisova Cave and our inability to specifically discern when these groups were dominant at the site may indicate the role scavenging played at Denisova Cave, particularly in regards to cave hyaenas and wolves taking advantage of the intensive hunting practices of Denisovans and Neanderthals^36^. Additionally, cut marked carnivore bones, particularly of red and polar foxes^52^, suggests hominins might have been targeting them for their fur. This practice is common in other Pleistocene sites in Russia, such as in the Kostenki region^53,54^.

The most notable difference between the morphological assemblage and the ZooMS-IDed component of Denisova Cave is the abundance of predators in comparison with herbivores. In the morphological assemblage of all three chambers, on average, predator groups account for 30.2% of all identified bones^29–34^, whereas in the ZooMS-IDed component predators account for only 7.6%. This discrepancy helps to inform us of the main factor influencing fragmentation rates at the site. Both the ZooMS ID-ed component and the morphological assemblage suggest that carnivores were the driving force behind the high rate of herbivore bone fragmentation. Given that the hominin bones recovered so far are similarly fragmented to herbivore bones (none exceed a few cm in length), we hypothesize that although humans were the main agent responsible for the accumulation and initial dismembering, butchering, and fragmentation of (most) herbivores in the cave, a secondary agent, that is carnivores such as hyenas, caused further processing and reduction in size of both animal and human bone deposited there. This hypothesis is largely in line with archaeological evidence for the site, where bones and teeth are frequently found with gnawing and digestion marks^32,33^.

### Faunal patterns and palaeoclimate

Recent work on building chronologies for the site using radiocarbon and optical dating^26,29^ has enabled the three chambers of Denisova Cave to be securely dated and cross-correlated. As a result, stratigraphic contexts from separate sections of the site can now be attributed to specific MIS stages and compared to one another. We identify several trends in the ZooMS-IDed component with reference to depositional age and the environmental conditions prevailing at the time. We note, for instance, that despite the wide variety of taxa the Cervidae/Gazella/Saiga ZooMS identification encompasses, they remain in relatively low abundance throughout the majority of the Pleistocene, except for the earliest layers which correspond to MIS 9–7 (337–191 ka). The majority of bones from this phase were excavated in layer 15 of the East Chamber which is attributed to MIS 7 and the Penultimate Interglacial, and coincides with the arrival of the first Denisovans to the site^27,35,50^. Layers 15 and 14 of the East Chamber have the highest abundance of stone tools of any other phase at Denisova Cave and a large proportion of humanly modified bone^27,35^. The morphological assemblage from these layers is dominated by the remains of Siberian roe deer (19%) and red deer (10%) that live in forest and grasslands^29,31^. Cervids were clearly favoured by these early Denisovans, an observation which remains in stark contrast to the faunal record of all later phases documented at the site.

Bison/Yak are the most abundant fauna for almost all subsequent (post-MIS 7) layers (Fig. 3; Supplementary Tables 1, 2, and 3). Steppe bison and Baikal yak are known to have favoured more open, steppe and forest-steppe environments^31,55^. Despite the expansion of forests during interglacial periods however, their remains continue to dominate the ZooMS-IDed component at 39% (MIS 3) and 31% (MIS 5) (Fig. 3; Supplementary Tables 1, 2, and 3). This suggests that rather than the environment being a driving force behind the abundance of fragmented *Bos* and *Bison* bones, predators, likely hominins, were preferentially targeting them, mirroring a trend identified in other Pleistocene assemblages analysed with ZooMS^22^.

With regards to predators in the ZooMS-IDed component, Canidae are the dominant group for the majority of the Pleistocene layers we studied. From MIS 9–4 their bones account for approximately 5% of the identified remains in our study. They are found alongside smaller numbers of other predators, including Felidae and *V. vulpes*. Crocuta/Panthera remains are first identified in the ZooMS-IDed component in layer 15 and they have been macroscopically identified in the morphological assemblage in layers covering the entire Pleistocene. The majority of these bones are probably cave hyaena, however, smaller numbers of snow leopard and Eurasian cave lion are also present throughout the stratigraphy^29–34^. Crocuta/Panthera become more common than Canidae during MIS 5–3, alongside archaeological evidence of intensive carnivore presence during the same period, particularly in the South Chamber (Fig. 3; Supplementary Tables 1, 2, and 3). Bear remains are identified throughout the Pleistocene layers of Denisova Cave both in the ZooMS-IDed component and the morphological assemblage. The extinct cave bears, *U. savini*, were mostly vegetarian, and used the cave for hibernation^32,33^.

### Animal size and impact on ZooMS-based fauna patterns

Body mass and size class, assigned based on previously published data^56^, probably play a disproportionate role in the number of taxa identified using ZooMS. For instance, Elephantidae, Bison/Yak, and Rhinocerotidae remains, all within body class size 6, are 3–5 times more abundant in the ZooMS-IDed component than the morphological dataset (Fig. 2). Bison/Yak remains are the most sensitive to this shift, accounting for only 6.3% of the morphological assemblage^29–34^ as opposed to 31% of the ZooMS-IDed component. A normalisation of the dataset based on body mass is not easy since it requires a high degree of confidence on other information, such as age and size of the targeted individuals, and carcass processing practices (e.g. transfer of specific body parts to the cave), which are unknown and/or fluctuate through time in the case of Denisova Cave.

In the case of mammoths, which were likely present in the Altai region in very small numbers and are not considered to have been a major contributor to hominin diet^57^, their bones are three times more abundant in the ZooMS identified megafauna (3.84% versus 1.08%). The only exception to this is the ratio of horse bones, for which no significant change in the percentage of their remains exists between the zooarchaeological dataset^29–34^ and the ZooMS-IDed component. With these factors in mind, any tentative predictive model for the abundance of large herbivores within a fragmented bone assemblage should consider both body class size and the likelihood that some taxa targeted by hominins were processed both in situ and at the cave site.

## Conclusions

The application of ZooMS at Denisova Cave highlights the potential of the method to elucidate early hunting practices and adaptation to new environments. The new data expands our understanding of the site’s faunal record and taphonomy, and the high success rate is illustrative of the potential of the method to other sites and regions with comparable biomolecular preservation and to material of the same or even greater antiquity, extending further back into the Middle Pleistocene.

Aside from its effectiveness in screening large numbers of bones for the identification of specific taxa, e.g. hominin fossils, ZooMS is complementary to traditional zooarchaeological practices as it allows a large part of the morphologically non-identifiable component to be diagnosed taxonomically. This is particularly true at Denisova Cave where traditional zooarchaeological analyses identified less than 5% of the excavated fauna versus ~ 80% identification success using ZooMS in this study.

The new data is also useful for elucidating the taphonomic history of the bones recovered from a site. In our case, this is shown by the differences in prey-predator ratios in the ZooMS-IDed versus the morphologically-identified assemblages. These differences reveal that the main influence in the fragmentation of herbivores/prey bones at Denisova was two-fold and operated at different levels. Hominin processing of carcasses was the main factor for the introduction, deposition and initial fragmentation of many of the herbivore and some of the carnivore bones at the site. This was followed by secondary and more extreme fragmentation, of both animal and human bones, as a result of predator scavenging and gnawing.

## Methods

Analysis was carried out at the ZooMS facility of the Department of Archaeology at the Max Planck Institute for the Science of Human History (MPI-SHH), Jena, Germany and the Manchester Institute of Biotechnology at the University of Manchester, UK. Samples analysed at the MPI-SHH followed published protocols using ammonium bicarbonate as the means of collagen extraction after which failed samples were re-attempted following acid soluble protocols^23,58–61^. Samples analysed at the University of Manchester followed published acid soluble protocols^60,61^. These protocols have all been optimised for the analysis of COL1 for taxonomic identification using ZooMS despite differences in initial protein extraction. The resulting spectra were screened for diagnostic markers using flexAnalysis 3.4 (Bruker Daltonics) and mMass software^62^. The spectra were compared against a reference library of known peptide markers^21,23,63^ which was informed further by previous zooarchaeological analysis carried out at Denisova Cave^29–34^.

Samples were analysed following established protocols. The ammonium bicarbonate buffer protocol^23,58,59^ involved sample rinsing in ammonium bicarbonate overnight and incubation for 1 h at 65 ℃. The supernatant was treated with 0.4 µg trypsin (Thermo Scientific Pierce ™ Trypsin Protease) and allowed to digest at 37 ℃ for 18 h. The incubated samples were concentrated and desalted using C18 ZipTips (Thermo Scientific Pierce™ C18 Tips) and eluted in a final solution of 50 µl of 50% acetonitrile and 0.1% TFA. 0.5 µl of the resulting solution was mixed with 0.5 µl of α cyano-4-hydroxycinnamic acid solution (10 mg/mL in 50% acetonitrile [ACN] and 0.1% trifluoroacetic acid [TFA]) and allowed to crystallise. The samples were analysed using a Bruker Autoflex Speed LRF MALDI ToF/ToF mass spectrometer.

Samples analysed using acid soluble protocols^25,60^ were demineralised in 0.6 M hydrochloric acid (HCl) for 18 h. The supernatant was removed into 30 kDa molecular weight cut-off (MWCO) ultrafilters and centrifuged at 3700 rpm for 1 h. The filtrate was then twice washed through with 500 μL of 50 mM ammonium bicarbonate (AmBic) and further centrifuged at 3700 rpm for half an hour after each treatment. The final residue was resuspended with additional AmBic (200 μL), half of which was removed to create a backup sample set before digestion. The remaining 100 μL was then treated with 0.2 μg trypsin (sequencing grade; Promega UK) and incubated at 37 °C for 18 h. The resulting solution was then diluted to 1:10 using TFA and mixed with a matrix solution of 1 μl of α-cyano-4-hydroxycinnamic acid solution (10 mg/mL in 50% ACN/0.1% TFA), allowed to crystallise and analysed using a Bruker Ultraflex II MALDI ToF/ToF mass spectrometer. For samples identified as ovicaprids, the tryptic peptide solution was further purified and fractionated using C18 Ziptips into 10% ACN and 50% ACN fractions (both in 0.1% TFA) and further analysed using MALDI as described above.

## Supplementary Information

Supplementary Dataset.

Supplementary Information.

## Data Availability

All ZooMS spectra for identified samples are available on Mendeley Data and are located in two databases. External Database 1: http://dx.doi.org/10.17632/5bwmbhs3fs.1. External Database 2: http://dx.doi.org/10.17632/bgm2k6gt3j.1.
