# Supplementary material for: Zooarchaeology through the lens of collagen fingerprinting at Denisova Cave
Source: Sci Rep. 2021 Jul 29;11:15457. doi: 10.1038/s41598-021-94731-2 (PMC8322063; doi:10.1038/s41598-021-94731-2)
Supplement: Supplementary file 2 — Supplementary Information. [file 41598_2021_94731_MOESM2_ESM.docx]

Supplementary Materials for

Zooarchaeology through the lens of collagen fingerprinting at Denisova Cave

Samantha Brown^1,2^*, Naihui Wang^2^, Annette Oertle^2^, Maxim B. Kozlikin^3^, Michael V. Shunkov^3^, Anatoly P. Derevianko^3^, Daniel Comeskey^4^, Blair Jope-Street^2^, Virginia L. Harvey^5,6^, Manasij Pal Chowdhury^5,6^, Michael Buckley^5,6^, Thomas Higham^4,7^, Katerina Douka^2,7^*

**Affiliations:**

^1^Institute for Scientific Archaeology, The University of Tübingen, Tübingen, Germany.

^2^Max Planck Institute for the Science of Human History, Jena, Germany.

^3^Institute of Archeology and Ethnography of the Siberian Branch of the Russian Academy of Sciences, Novosibirsk, Russia.

^4^Oxford Radiocarbon Accelerator Unit, RLAHA, University of Oxford, Oxford OX13QY, UK.

^5^The University of Manchester, Department of Earth and Environmental Sciences, School of Natural Sciences, Manchester, M13 9PL, UK

^6^Manchester Institute of Biotechnology, The University of Manchester, Manchester, M1 7DN, UK

^7^Department of Evolutionary Anthropology, Faculty of Life Sciences, University of Vienna, Vienna, Austria

^*^Correspondence to: samantha.brown@uni-tuebingen.de, [douka@shh.mpg.de](mailto:douka@shh.mpg.de)

**This PDF file includes:**

Supplementary Text

Supplementary Tables 1-4

**Other Supplementary Materials for this manuscript include the following:**

Supplementary Database 1: ZooMS Results Table

External Database 1: ZooMS spectra files

External Database 2: ZooMS spectra files

Supplementary Text

Several important qualifying factors are necessary in understanding the ZooMS results presented in this study. Small vertebrates, for instance, are unlikely to be identified through the use of ZooMS, in part because the current established ZooMS reference library largely excludes several taxa present in the Altai, including the Sciuridae, Dipodidae, and Spalacidae families, and in part because of our preferential selection of mammal bones larger than 2 cm. The spiral horned antelope (*Spirocerus kiakhtensis*) is the only medium-large vertebrate which does not currently have markers in the ZooMS reference library. *S. kiakhtensis* were found throughout Northern and Central Asia until the Late Pleistocene[^1^](https://paperpile.com/c/BL5yjP/NG9b6) and appear in small numbers at Denisova Cave.[^2–8^](https://paperpile.com/c/BL5yjP/sET8p+3eNcw+NX4CF+l0odE+1vdLI+2J3Ao+KMq0Z) Until a full proteomic analysis of securely attributed *S.  kiakhtensis* remains is completed it is not clear whether any collagen peptide markers exist for this taxon. 26 samples did not match taxa in the ZooMS reference library and have been reported in our results as being “Unknown” (Table 1; Supplementary Table 2, 3, 4).

Further, the confident separation of key taxa is not always possible. Ovis/Capra for instance can be differentiated on the basis of their COL1ɑ2 484 - 498 peptide. However, this peptide is often lost as a result of collagen degradation, a particular problem when working with Pleistocene fossils. Where possible we have attempted to distinguish between Ovis and Capra or used Ovis/Capra when the diagnostic marker was missing. Ambiguity also surrounds the differentiation of Crocuta and Panthera taxa. It is theoretically possible to separate the two groups using the COL1ɑ2 793 – 816 peptide marker however this is another peptide which is easily lost as a result of collagen degradation. Additionally, the marker has not been reliably identified for all Panthera groups studied [^9^](https://paperpile.com/c/BL5yjP/0e9C3) and so, to ensure a measure of confidence in our data, we have not attempted to differentiate between Crocuta and Panthera.

ZooMS Analysis Results

We analysed 6,288 samples from most archaeological layers of the East Chamber. These include: layer 9.2 (n= 89), 9.3 (n= 196), 11.2 (n= 620), 11.3 (n= 95), 11.4 (n= 353), 12 (n= 2,291), 12.3 (n= 165), 13 (n= 28), 14 (n= 1381), 15 (n= 967), 17.1 (n= 53) (Supplementary Figure 1). An additional 50 samples were excavated at the interface between layers 12.1 and 12.2. 82% of the samples analysed from the East Chamber could be assigned to a specific ZooMS taxon and an additional 2% of samples produced low quality spectra which could only be identified to family or order. 16% of samples failed ZooMS analysis meaning that overall, the East Chamber had the lowest percentage of samples which failed to produce enough collagen for taxonomic identification (Supplementary Table 2; Supplementary Data 1).

We analysed 1,143 samples from the Main Chamber, the majority of which were excavated during the 2016 season. These come from Middle Palaeolithic and Upper Palaeolithic contexts, layers 9.1 (n= 36), 9.2 (n=1), 9.3 (n= 82), 11 (n= 11), 11.1 (n= 115), 11.2 (n= 181), 11.3 (n= 139), 11.4 (n= 542). An additional 36 samples were found at the interface between layers 9.3 and 11.2 (n= 6) as well as layers 11.4 and 12 (n= 30) (Supplementary Figure 1). Overall, a high level of identification was possible for this layer with 75% of samples assigned to a specific ZooMS taxon and an additional 7% of samples producing low quality spectra which could be identified to family or order. 17% of samples failed to produce enough collagen for taxonomic identification, the majority of which were excavated from layers 9.1 and 9.3 (Supplementary Table 3; Supplementary Data 1).

We analysed 822 samples from the South Chamber, the majority derive from recent excavations of layers 11 (n= 461) and 12 (358) (Supplementary Figure 1). An additional three samples for layer 21 were analysed, one failed and the other two were identified as Bison/Yak and Cervidae/Gazella/Saiga. 65% of samples could be identified using ZooMS and an additional 7% of samples produced low quality spectra and could not be assigned to a single ZooMS taxon. The South Chamber produced the highest number of failed samples, with 28% of samples failing to produce enough collagen for taxonomic identification (Supplementary Table 4; Supplementary Data 1).

Supplementary Tables

Supplementary Table 1: Species identified in the morphological datasets from Denisova Cave which have been grouped under the most precise ZooMS identification taxon possible, their “ZooMS taxon”. Taxa and body class size information compiled from previously published data[^2–8,10^](https://paperpile.com/c/BL5yjP/sET8p+3eNcw+NX4CF+l0odE+1vdLI+2J3Ao+KMq0Z+9etb7).

| **ZooMS taxon** | | **Body class size** | | **Family** | | **Genus** | | **Species** |
| --- | --- | --- | --- | --- | --- | --- | --- | --- |
| Leporidae | | - | | Leporidae | | Lepus | | *Lepus tanaiticus* |
|  |  | 3.2 | | Leporidae | | Lepus | | *Lepus tolai* |
| Ochotonidae | | - | | Ochotonidae | | Ochotona | | *Ochotona* sp*.* |
| Cricetidae | | 2.71 | | Cricetidae | | Cricetus | | *Cricetus* sp*.* |
| Castoridae | | 4.28 | | Castoridae | | Castor | | *Castor fiber* |
| Vulpes vulpes | | 3.77 | | Canidae | | Vulpes | | *Vulpes vulpes* |
| Canidae | | 4.23 | | Canidae | | Canis | | *Canis lupus* |
|  |  | - | | Canidae | | Vulpes | | *Vulpes corsak* |
|  |  | 4.11 | | Canidae | | Cuon | | *Cuon alpinus* |
|  |  | 3.54 | | Canidae | | Vulples | | *Alopex lagopus* |
| Ursidae | | 5.14 | | Ursidae | | Ursus | | *Ursus (Spelaearctos) savini* |
|  |  |  |  |  |  |  |  |  |
| Mustelidae | - | | Mustelidae | | Martes | | *Martes zibellina* | |
|  | - | | Mustelidae | | Gulo | | *Gulo gulo* | |
|  | - | | Mustelidae | | Mustela | | *Mustela erminea* | |
|  | - | | Mustelidae | | Mustela | | *Mustela nivalis* | |
|  | - | | Mustelidae | | Mustela | | *Mustela sibirica* | |
|  | - | | Mustelidae | | Mustela | | *Mustela altaica* | |
|  | - | | Mustelidae | | Mustela | | *Mustela eversmanni* | |
| Crocuta/Panthera | 4.8 | | Hyaenidae | | Crocuta | | *Crocuta crocuta spelaea* | |
|  | - | | Felidae | | Panthera | | *Panthera spelaea* | |
|  | 4.65 | | Felidae | | Panthera | | *Uncia uncia* | |
| Felidae | 4.25 | | Felidae | | Lynx | | *Lynx lynx* | |
|  | 3.54 | | Felidae | | Otocolobus | | *Felis manul* | |
| Elephantidae | 6.74 | | Elephantidae | | Mammuthus | | *Mammuthus primigenius* | |
| Equidae | 5 | | Equidae | | Equus | | *Equus (Equus) ferus* | |
|  | 5 | | Equidae | | Equus | | *Equus (Sussemionus) ovodovi* | |
|  | 5 | | Equidae | | Equus | | *Equus hydruntinus* | |
| Rhinocerotidae | 6 | | Rhinocerotidae | | Coelodonta | | *Coelodonta antiquitatis* | |
| Rangifer | 4.93 | | Cervidae | | Rangifer | | *Rangifer tarandus* | |
| Cervidae | - | | Cervidae | | Capreolus | | *Capreolus pygargus* | |
| Cervidae/Gazella/Saiga | 5.19 | | Cervidae | | Cervus | | *Cervus elaphus* | |
|  | 5.9 | | Cervidae | | Megaloceros | | *Megaloceros giganteus* | |
|  | 5.55 | | Cervidae | | Alces | | *Alces* cf. *alces* | |
|  | 4.46 | | Bovidae | | Saiga | | *Saiga tatarica borealis* | |
|  | 4.45 | | Bovidae | | Gazella | | *Procapra gutturosa* | |
|  | 4 | | Bovidae | |  | | *Gazella / Saiga* | |
| Bison/Yak | 5.95 | | Bovidae | | Bison | | *Bison priscus* | |
|  | - | | Bovidae | | Bos | | *Poёphagus mutus* | |
| Capra | 5.11 | | Bovidae | | Capra | | *Capra sibirica* | |
| Ovis | 5.26 | | Bovidae | | Ovis | | *Ovis ammon* | |
| Unknown | - | | Sciuridae | | Pteromys | | *Pteromys volans* | |
|  | 2.2 | | Bovidae | | Spirocerus | | *Spirocerus kiakhtensis* | |
|  | 1.93 | | Sciuridae | | Spermophilus | | *Spermophilus sp.* | |
|  | - | | Sciuridae | | Marmota | | *Marmota baibacina* | |
|  | 2.37 | | Dipodidae | | Allactaga | | *Allactaga sp.* | |
|  | 2.35 | | Spalacidae | | Myospalax | | *Myospalax myospalax* | |

Supplementary Table 2: Number of samples analysed using ZooMS for the East Chamber of Denisova Cave separated by layer. Marine oxygen isotope stage (MIS) attribution is based on previously published data.^6, 11^

| **ZooMS Taxon** | **Stratigraphic layers for the East Chamber of Denisova Cave** | | | | | | | | | | | | |
| --- | --- | --- | --- | --- | --- | --- | --- | --- | --- | --- | --- | --- | --- |
|  | **9.2**  **(MIS 3)** | **9.3**  **(MIS 3)** | **11.2**  **(MIS 4-3)** | **11.3**  **(MIS 5-4)** | **11.4**  **(MIS 5)** | **12**  **(MIS 6-5)** | **12.1-12.2**  **(MIS 6-5)** | **12.3**  **(MIS 6-5)** | **13**  **(MIS 6)** | **14**  **(MIS 7-6)** | **15**  **(MIS 7)** | **17.1**  **(MIS 9-8)** | **Grand Total** |
| Bird |  |  | 1 | 1 |  | 5 |  | 1 |  | 1 |  |  | 9 |
| Bison/Yak | 41 | 54 | 206 | 20 | 81 | 710 | 16 | 36 | 13 | 289 | 162 | 1 | 1629 |
| Canidae |  |  | 6 | 6 | 13 | 58 | 2 | 4 |  | 33 | 16 | 2 | 140 |
| Capra | 1 | 2 | 27 | 15 | 16 | 101 |  | 12 | 1 | 19 | 14 | 1 | 209 |
| Cervidae | 1 |  | 1 |  | 7 | 2 | 1 |  |  | 6 |  |  | 18 |
| Cervidae/Gazella/Saiga | 4 | 19 | 61 | 18 | 48 | 292 | 5 | 11 | 1 | 292 | 436 | 2 | 1189 |
| Crocuta/Panthera | 2 | 4 | 8 | 3 | 2 | 70 | 1 | 1 |  | 7 | 1 |  | 99 |
| Elephantidae |  | 3 | 9 |  | 14 | 106 | 1 | 19 | 1 | 21 | 7 |  | 181 |
| Equidae | 5 | 15 | 44 | 4 | 35 | 257 | 4 | 20 | 4 | 177 | 100 | 4 | 669 |
| Felidae |  |  |  |  |  |  |  |  |  | 5 | 2 |  | 7 |
| Hominin |  |  |  |  |  | 2 |  |  |  |  | 4 |  | 6 |
| Leporidae |  |  |  | 1 |  | 1 |  |  |  |  |  |  | 2 |
| Muridae |  |  |  |  |  | 1 |  |  |  | 1 |  |  | 2 |
| Mustelidae |  |  |  |  |  | 1 | 1 |  |  |  |  |  | 2 |
| Ovis | 1 | 1 | 16 | 13 | 9 | 91 |  | 6 | 1 | 7 | 2 |  | 147 |
| Ovis/Capra | 3 | 13 | 15 |  | 11 | 159 | 6 | 14 | 3 | 56 | 22 |  | 302 |
| Rangifer |  | 1 | 2 |  |  | 26 |  | 3 |  | 1 | 1 |  | 34 |
| Rhinocerotidae | 1 | 5 | 32 | 8 | 16 | 176 |  | 16 | 1 | 110 | 36 |  | 403 |
| Ursidae |  | 1 | 8 |  | 8 | 67 |  | 2 |  | 28 | 5 | 4 | 123 |
| Vulpes vulpes |  |  | 1 | 1 | 4 | 9 |  | 2 |  | 1 |  | 1 | 19 |
|  | | | | | | | | | | | | | |
| Capra/Rangifer |  | 1 |  |  | 4 | 1 |  |  |  | 6 |  |  | 12 |
| Cervidae/Gazella/Saiga/  Equidae |  | 2 | 2 |  |  | 2 |  |  |  | 5 | 4 |  | 15 |
| Crocuta/Panthera/  Mustelidae |  |  | 1 |  | 3 | 4 |  |  |  | 2 | 1 |  | 11 |
| Felidae/Crocuta/  Panthera |  |  |  |  |  |  |  |  |  |  | 1 |  | 1 |
| Felidae/Crocuta/  Panthera/Mustelidae |  |  | 2 |  |  |  |  |  |  |  |  |  | 2 |
| Felidae/Ursidae |  |  | 4 |  | 1 | 1 |  |  |  | 7 | 3 | 2 | 18 |
| Ovis/Capra/Cervidae/  Gazella/Saiga |  | 2 | 1 |  | 4 | 4 |  |  |  | 14 |  |  | 25 |
| Ovis/Capra/Rangifer |  |  | 1 |  | 1 |  |  |  |  | 1 |  | 1 | 4 |
| Ovis/Cervidae/Gazella/  Saiga |  |  | 3 |  |  |  |  |  |  | 18 | 4 |  | 25 |
|  | | | | | | | | | | | | | |
| **Unknown** |  |  | **1** |  | **3** | **7** |  |  | **1** | **4** | **2** | **1** | **19** |
| **Success** | **59** | **123** | **452** | **90** | **280** | **2153** | **37** | **147** | **26** | **1111** | **823** | **19** | **5320** |
| **Failed** | **30** | **73** | **168** | **5** | **73** | **138** | **13** | **18** | **2** | **270** | **144** | **34** | **968** |
| **Grand Total** | **89** | **196** | **620** | **95** | **353** | **2291** | **50** | **165** | **28** | **1381** | **967** | **53** | **6288** |

Supplementary Table 3: Number of individual samples analysed using ZooMS for the Main Chamber of Denisova Cave separated by layer. Marine oxygen isotope stages (MIS) attribution is based on previously published data^6, 11^.

| **ZooMS Taxon** | **Stratigraphic layers for the Main Chamber of Denisova Cave** | | | | | | | | | | | |
| --- | --- | --- | --- | --- | --- | --- | --- | --- | --- | --- | --- | --- |
|  | **9.1**  **(MIS 3-2)** | **9.2**  **(MIS 3-2)** | **9.3**  **(MIS 3-2)** | **9.3/11.2**  **(MIS 3)** | **11**  **(MIS 3)** | **11.1**  **(MIS 3)** | **11.2**  **(MIS 3)** | **11.3**  **(MIS 3)** | **11.4**  **(MIS 3)** | **11.4/12**  **(MIS 5-3)** | **Grand Total** |  |
| Bird |  |  |  |  |  |  |  | 1 |  |  | 1 |  |
| Bison/Yak | 4 | 1 | 19 | 1 | 2 | 49 | 46 | 37 | 169 | 9 | 337 |  |
| Canidae |  |  | 2 |  |  | 1 | 2 | 3 | 4 |  | 12 |  |
| Capra |  |  | 1 |  |  | 2 | 18 | 14 | 39 | 1 | 75 |  |
| Cervidae/Gazella/Saiga | 1 |  | 4 |  |  | 4 | 6 | 4 | 15 | 1 | 35 |  |
| Crocuta/Panthera |  |  | 2 |  |  | 5 | 5 | 3 |  |  | 21 |  |
| Elephantidae | 1 |  | 1 |  |  | 5 | 9 | 9 | 15 |  | 40 |  |
| Equidae | 4 |  | 3 |  | 2 | 4 | 21 | 12 | 73 | 4 | 123 |  |
| Felidae |  |  |  |  |  |  |  |  | 1 |  | 1 |  |
| Hominin | 1 |  |  |  |  |  |  |  |  |  | 1 |  |
| Ovis | 2 |  | 6 | 2 | 3 | 9 | 15 | 19 | 48 | 4 | 108 |  |
| Ovis/Capra | 1 |  | 6 |  | 2 | 11 | 7 | 11 | 26 | 3 | 67 |  |
| Rangifer |  |  |  |  |  |  |  |  | 3 |  | 3 |  |
| Rhinocerotidae | 1 |  | 6 | 1 | 1 | 10 | 20 | 3 | 54 | 2 | 98 |  |
| Ursidae |  |  | 2 | 1 |  | 2 | 3 | 4 | 8 |  | 20 |  |
|  | | | | | | | | | | | | |
| Capra/Rangifer |  |  |  |  |  |  | 1 |  | 1 |  | 2 |  |
| Crocuta/Panthera/Mustelidae |  |  |  |  |  |  | 1 |  | 4 |  | 5 |  |
| Equidae/Rhinocerotidae |  |  |  |  |  | 1 |  |  |  |  | 1 |  |
| Felidae/Ursidae |  |  |  |  |  |  | 1 |  | 3 |  | 4 |  |
| Ovis/Capra/Cervidae/  Gazella/Saiga |  |  |  |  |  |  |  | 1 | 1 |  | 2 |  |
| Ovis/Cervidae/Gazella/Saiga | 1 |  |  |  |  |  | 1 | 1 |  |  | 3 |  |
|  | | | | | | | | | | | | |
| **Unknown** |  |  |  |  |  |  |  | **1** |  | **2** | **3** |  |
| **Success** | **16** | **1** | **52** | **5** | **9** | **102** | **148** | **120** | **446** | **26** | **926** |  |
| **Failed** | **20** |  | **30** | **1** | **1** | **12** | **26** | **17** | **72** | **4** | **183** |  |
| **Grand Total** | **36** | **1** | **82** | **6** | **11** | **115** | **181** | **139** | **542** | **30** | **1143** |  |

Supplementary Table 4: Number of samples analysed using ZooMS for the South Chamber of Denisova Cave separated by layer. Marine oxygen isotope stages (MIS) attribution is based on previously published data^6, 11^. Reliable age estimations have not yet been achieved for layer 21, therefore no corresponding MIS is listed.

| **ZooMS Taxon** | **Stratigraphic layers for the South Chamber of Denisova Cave** | | | |
| --- | --- | --- | --- | --- |
|  | **11**  **(MIS 5-3)** | **12**  **(MIS 5-3)** | **21** | **Grand Total** |
| Bison/Yak | 91 | 129 | 1 | 221 |
| Canidae | 5 | 3 |  | 8 |
| Capra | 18 | 12 |  | 30 |
| Cervidae/Gazella/Saiga | 14 | 17 | 1 | 32 |
| Crocuta/Panthera | 12 | 7 |  | 19 |
| Elephantidae | 12 | 23 |  | 35 |
| Equidae | 27 | 31 |  | 58 |
| Ovis | 18 | 22 |  | 40 |
| Ovis/Capra | 35 | 19 |  | 54 |
| Rangifer | 1 |  |  | 1 |
| Rhinocerotidae | 25 | 23 |  | 48 |
| Ursidae | 29 | 4 |  | 33 |
|  | | | | |
| Capra/Rangifer |  | 3 |  | 3 |
| Cervidae/Gazella/Saiga/Equidae |  | 1 |  | 1 |
| Crocuta/Panthera/Mustelidae | 1 | 3 |  | 4 |
| Felidae/Ursidae | 1 |  |  | 1 |
| Ovis/Capra/Rangifer | 2 |  |  | 2 |
|  | | | | |
| **Unknown** | **2** | **1** |  | **3** |
| **Success** | **294** | **298** | **2** | **594** |
| **Failed** | **168** | **60** | **1** | **229** |
| **Grand Total** | **461** | **358** | **3** | **822** |

**Supplementary Figures:**

**
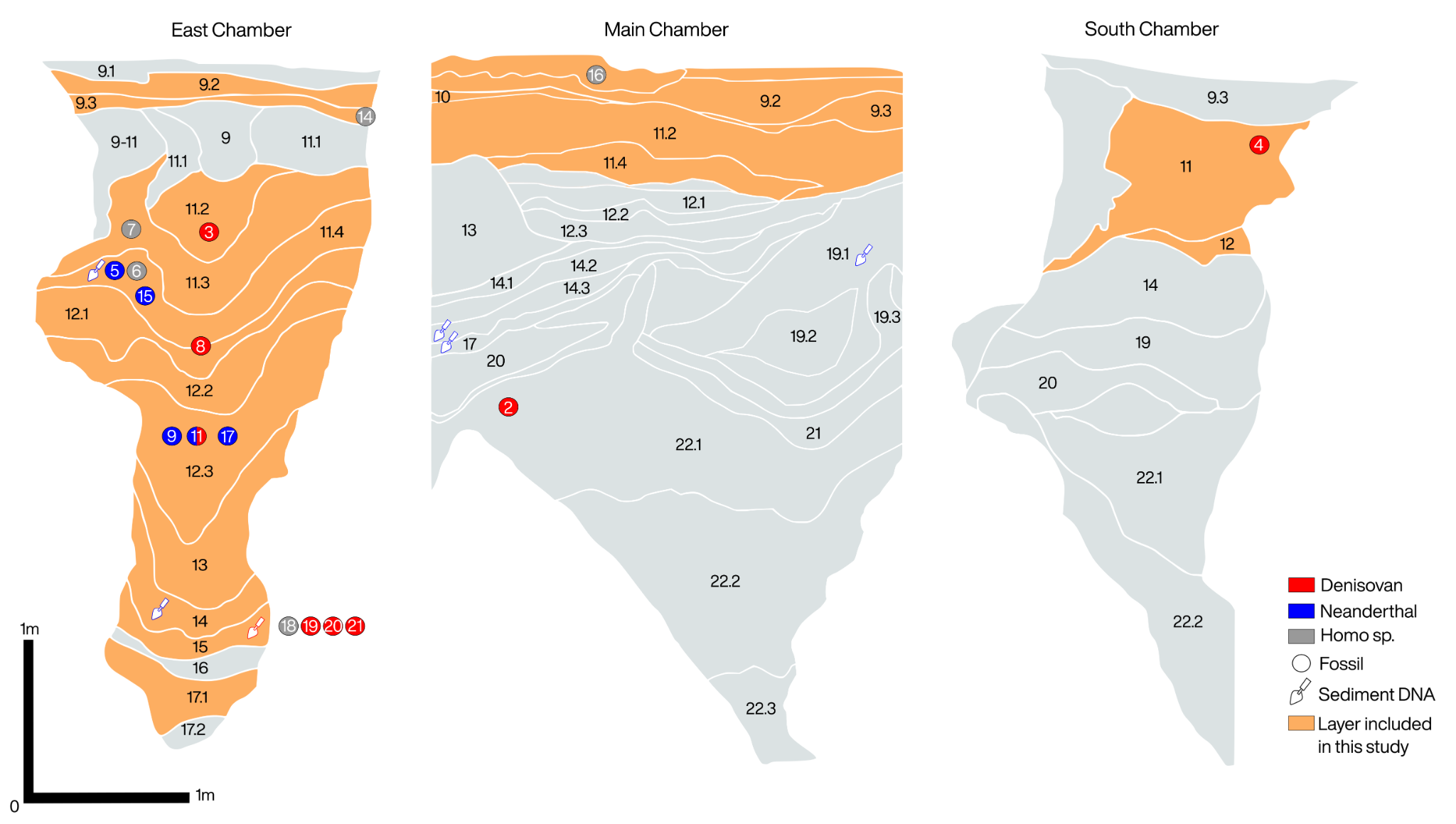
**

Supplementary Figure 1: Stratigraphy for the three chambers of Denisova Cave. Layers studied as part of this research are highlighted in orange. Hominins identified in previous research are shown in circles[^11–18^](https://paperpile.com/c/BL5yjP/AuNIe+HbT6r+0YjRJ+nyMFx+zNti8+SZA6+BEXG8+d23E) and layers for which eDNA analysis has been carried out are indicated.[^19^](https://paperpile.com/c/BL5yjP/749w)

**External Database:**

All data has been uploaded to Mendeley Data. ZooMS spectra for each of the identified samples can be accessed from two databases;

External Database 1: <http://dx.doi.org/10.17632/5bwmbhs3fs.1>

External Database 2: http://dx.doi.org/10.17632/bgm2k6gt3j.1

Supplementary References

1. [Kalmykov, N. P., Kobylkin, D. V., Grigoryeva, M. A. & Chernykh, V. N. Validity of the spiral-horned antelope species of the genus Spirocerus (Mammalia, Artiodactyla) in Central Asia. *Dokl. Biol. Sci.* **457**, 233–235 (2014).](http://paperpile.com/b/BL5yjP/NG9b6)

2. [Morley, M. W. *et al.* Hominin and animal activities in the microstratigraphic record from Denisova Cave (Altai Mountains, Russia). *Sci. Rep.* **9**, 13785 (2019).](http://paperpile.com/b/BL5yjP/sET8p)

3. [Vasiliev, S. K., Shunkov, M. V. & Kozlikin, M. B. Preliminary Results for the Balance of Megafauna from Pleistocene Layers of the East Gallery, Denisova Cave. *Problems of Archaeology, Ethnography, and Anthropology of Siberia and Adjacent Territories* **19**, 32–38 (2013).](http://paperpile.com/b/BL5yjP/3eNcw)

4. [Vasiliev S.K., Shunkov M.V., Kozlikin M.B. Megafaunal Remains from the Eastern Chamber of Denisova Cave and Problems of Reconstructing the Pleistocene Environments in the Northwestern Altai. *Problems of Archaeology, Ethnography, Anthropology of Siberia and Neighboring Territories* **XXIII**, (2017).](http://paperpile.com/b/BL5yjP/NX4CF)

5. [S.K. Vasiliev, M.B. Kozlikin, M.V. Shunkov. Megafaunal Remains from the Upper Portion of Pleistocene Deposits in South Chamber of Denisova Cave. *Problems of Archaeology, Ethnography, Anthropology of Siberia and Neighboring Territories* **569**, (2018).](http://paperpile.com/b/BL5yjP/l0odE)

6. [Jacobs, Z. *et al.* Timing of archaic hominin occupation of Denisova Cave in southern Siberia. *Nature* **565**, 594–599 (2019).](http://paperpile.com/b/BL5yjP/1vdLI)

7. [Vasiliev, S. K. & Shunkov, M. V. Large Pleistocene Mammals in the Southern Gallery of Denisova Cave. *Problems of Archaeology, Ethnography, Anthropology of Siberia and Neighboring Territories* **XV**, 63–69 (2009).](http://paperpile.com/b/BL5yjP/2J3Ao)

8. [Agadjanian, A. K. & Serdyuk, N. V. The history of mammalian communities and paleogeography of the Altai Mountains in the Paleolithic. *Paleontol. J.* **39**, 645–821 (2005).](http://paperpile.com/b/BL5yjP/KMq0Z)

9. [Welker, F. *et al.* Palaeoproteomic evidence identifies archaic hominins associated with the Châtelperronian at the Grotte du Renne. *Proc. Natl. Acad. Sci. U. S. A.* **113**, 11162–11167 (2016).](http://paperpile.com/b/BL5yjP/0e9C3)

10. [Smith, F. A. *et al.* Body mass of late Quaternary mammals. *Ecology* **84**, 3402 (2003).](http://paperpile.com/b/BL5yjP/9etb7)

11. [Brown, S. *et al.* Identification of a new hominin bone from Denisova Cave, Siberia using collagen fingerprinting and mitochondrial DNA analysis. *Sci. Rep.* **6**, 23559 (2016).](http://paperpile.com/b/BL5yjP/AuNIe)

12. [Douka, K. *et al.* Age estimates for hominin fossils and the onset of the Upper Palaeolithic at Denisova Cave. *Nature* **565**, 640–644 (2019).](http://paperpile.com/b/BL5yjP/HbT6r)

13. [Brown, S. *et al.* Earliest evidence for Denisovans identified using peptide mass fingerprinting and mitochondrial DNA analysis.](http://paperpile.com/b/BL5yjP/0YjRJ)

14. [Prüfer, K. *et al.* The complete genome sequence of a Neanderthal from the Altai Mountains. *Nature* **505**, 43–49 (2014).](http://paperpile.com/b/BL5yjP/nyMFx)

15. [Reich, D. *et al.* Genetic history of an archaic hominin group from Denisova Cave in Siberia. *Nature* **468**, 1053–1060 (2010).](http://paperpile.com/b/BL5yjP/zNti8)

16. [Slon, V. *et al.* The genome of the offspring of a Neanderthal mother and a Denisovan father. *Nature* **561**, 113–116 (2018).](http://paperpile.com/b/BL5yjP/SZA6)

17. [Mednikova, M. B. A proximal pedal phalanx of a Paleolithic hominin from denisova cave, Altai. *Archaeology, Ethnology and Anthropology of Eurasia* **39**, 129–138 (2011).](http://paperpile.com/b/BL5yjP/BEXG8)

18. [Sawyer, S. *et al.* Nuclear and mitochondrial DNA sequences from two Denisovan individuals. *Proc. Natl. Acad. Sci. U. S. A.* **112**, 15696–15700 (2015).](http://paperpile.com/b/BL5yjP/d23E)

19. [Slon, V. *et al.* Neandertal and Denisovan DNA from Pleistocene sediments. *Science* **356**, 605–608 (2017).](http://paperpile.com/b/BL5yjP/749w)
